# Structural, Thermal and Dielectric Properties of Low Dielectric Permittivity Cordierite-Mullite-Glass Substrates at Terahertz Frequencies

**DOI:** 10.3390/ma14144030

**Published:** 2021-07-19

**Authors:** Beata Synkiewicz-Musialska, Dorota Szwagierczak, Jan Kulawik, Norbert Pałka, Przemysław Piasecki

**Affiliations:** 1 Łukasiewicz Research Network—Institute of Microelectronics and Photonics, Kraków Division, ul. Zabłocie 39, 30-701 Kraków, Poland; dorota.szwagierczak@imif.lukasiewicz.gov.pl (D.S.); jan.kulawik@imif.lukasiewicz.gov.pl (J.K.); 2 Institute of Optoelectronics, Military University of Technology, ul. gen. W. Urbanowicza 2, 00-908 Warszawa, Poland; norbert.palka@wat.edu.pl; 3 Institute of Radioelectronics and Multimedia Technology, Warsaw University of Technology, ul. Nowowiejska 15/19, 00-665 Warszawa, Poland; p.piasecki@pitradwar.com; 4 PIT-RADWAR S.A., ul. Poligonowa 30, 04-051 Warszawa, Poland

**Keywords:** cordierite, mullite, glass–ceramic composites, low dielectric permittivity, dielectric properties, terahertz frequencies

## Abstract

Glass–ceramic composites containing cordierite, mullite, SiO_2_ glass and SiO_2_-B_2_O_3_-Al_2_O_3_-BaO-ZrO_2_ glass were fabricated in a process comprising solid state synthesis, milling, pressing and sintering. Thermal behavior, microstructure, composition and dielectric properties in the Hz-MHz, GHz and THz ranges were examined using a heating microscope, differential thermal analysis, thermogravimetry, scanning electron microscopy, energy dispersive spectroscopy, X-ray diffraction analysis, impedance spectroscopy, transmission method and time domain spectroscopy (TDS). The obtained substrates exhibited a low dielectric permittivity of 4.0–4.8. Spontaneously formed closed porosity dependent on the sintering conditions was considered as a factor that decreased the effective dielectric permittivity.

## 1. Introduction

The use of substrate materials that display a low dielectric permittivity and a low dielectric loss is crucial for attaining high signal propagation speed and high frequency selectivity of modern electronic circuits that operate at very high frequencies [1,2,3,4,5]. A combination of low dielectric permittivity polycrystalline materials and glasses rich in SiO_2_/B_2_O_3_ can result in glass–ceramic materials demonstrating the required microwave properties. Introducing controlled closed porosity is also a possible way to decrease the dielectric permittivity.

Cordierite and mullite belong to the group of the most popular and cheap ceramic materials characterized by a low dielectric permittivity. Low dielectric permittivity (4.8–6 for cordierite and 6–7.2 for mullite), low dielectric loss, low thermal expansion coefficient and good mechanical strength are the desired properties of these materials in microwave applications [6,7,8,9,10,11,12,13,14,15,16,17,18,19,20,21,22,23,24,25,26]. The disadvantage of their high sintering temperatures can be overcome by adding low melting glasses.

Glass–ceramic materials which can combine the advantages of both glasses and ceramics and are suitable for LTCC (low temperature cofired ceramics) multilayer technology have attracted great attention in recent years [27]. Liquid phase assisted sintering is typically responsible for the densification of glass–ceramic composites containing nonreactive glasses [28,29,30,31]. Initially, rearrangement of ceramic grains and rearrangement of glass occur, the latter related to capillary forces in pore channels [30]. Small wetting angles (below 90°) between solid particles and glass determine the effectiveness of this densification step. Finally, residual porosity is eliminated by the viscous flow of glass with sufficiently low viscosity. The use of glass with a higher viscosity can entail an increase in sintering temperature, prolonged firing time or lowering of the ceramic fraction to obtain well densified glass–ceramic material [30].

This paper presents a broad characterization of the structural, thermal and dielectric properties of cordierite-mullite-glass substrates containing a significant fraction of spontaneously formed closed porosity. Measurements of the dielectric properties at very high frequencies and spreading the characterization range into the THz range, are important in view of the trend for a permanent increase in the operating frequencies of telecommunication devices.

## 2. Materials and Methods

To prepare the glass–ceramic powder, a mixture composed of 30 wt.% cordierite Mg_2_Al_4_Si_5_O_18_, 10 wt.% mullite Al_6_Si_2_O_13_, 30 wt.% SiO_2_ glass and 30 wt.% glass C (SiO_2_ 30.5 wt.%, B_2_O_3_ 23.1 wt.%, Al_2_O_3_ 3.6 wt.%, BaO 38 wt.%, ZrO_2_ 4.8 wt.%) was milled for 8 h in isopropyl alcohol using agate grinding media and a ball mill (Pulverisette 5, Fritsch, Germany). With the exception of laboratory made glass C, all starting materials were high purity (>99.9%) Sigma Aldrich (St. Louis, MO, USA) products.

After the powder had been dried and a granulate had been made with polyvinyl alcohol, glass–ceramic pellets were uniaxially pressed and sintered in the temperature range 950–1050 °C for 2–12 h. Several firing profiles were tested corresponding to different heating rates (3–8 min/°C) and cooling rates. Furthermore, holding steps were introduced at 500 °C for 1 h to burnout the organic binder and at 870 °C for 6 h to enable the glass C softening and its viscous flow. The changes in the shape and dimensions of the samples during heating between 20 and 1330 °C were recorded using a heating microscope (Leitz, Germany). These observations allowed the softening and melting temperatures of the glasses, the sintering temperatures of the glass–ceramic composites, and the shrinkage of the samples to be determined. Furthermore, indicating the temperature ranges in which violent reactions with gas evolution occurred was possible. Differential thermal analysis (DTA) and thermogravimetric (TG) studies between 20 and 1000 °C were carried out using a thermal analyzer (STA 449 F3, Netzsch, Selb, Germany).

The phase composition of the glass–ceramic composites was analyzed by the XRD method using Cu K*_α_*_1_ radiation (PANalytical, Almelo, The Netherlands). The microstructure and elemental composition of the sintered glass–ceramic substrates were investigated using scanning electron microscopy and X-ray energy dispersive spectroscopy (FEI Nova Nano SEM 200 with EDAX Genesis system, Hillsboro, OR, USA). The bulk density of the samples was examined by the Archimedes method and the relative density was determined based on the bulk and theoretical densities.

The dielectric properties within the frequency range 100 Hz to 2 MHz and within the temperature range −30 to 150 °C were investigated using the impedance spectroscopy method (QuadTech 7600 LCR meter, Roslyn Heights, NY, USA). The AC test voltage was set at 1 V. The samples were placed in climatic Vötsch chambers with temperature control ±1 °C.

The characterization of dielectric properties at room temperature in the 90–140 GHz band was performed by the transmission method using a laboratory quasi-optical measurement setup (Institute of Radioelectronics and Multimedia Technology, Warsaw University of Technology, Poland) with two horn antennas and two off-axis parabolic mirrors [32]. The determination of dielectric parameters was based on the measured complex S-parameters obtained for two cases: without a sample and with a sample placed in the optical path between the mirrors. To restrict the error of dielectric permittivity estimation, the front and back planes of the samples were grinded to ensure surfaces parallel to each other. The dielectric properties of the material were extracted using special numerical algorithms implemented in Matlab.

The dielectric characteristics at room temperature in the 0.15–1.5 THz range were gathered using the time domain spectroscopy method (TDS). The setup for these measurements (TPS Spectra 3000, TeraView, Cambridge, UK) is destined for transmission and attenuated total reflection measurements. It comprises a THz pulsed spectrometer with a femtosecond laser, photoconductive antennas and a delay line. It can generate and detect about 0.5 ps long electromagnetic pulse in the range of 0.1–3.5 THz with a 10 GHz resolution.

## 3. Results and Discussion

### 3.1. Heating Behavior, Microstructure, Elemental and Phase Composition

**Figure 1:**
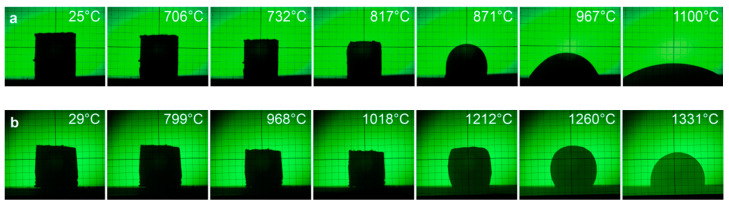
Selected images from a heating microscope: (**a**) glass C, (**b**) 30 wt.% glass C-30 wt.% SiO_2_ glass-30 wt.% cordierite-10 wt.% mullite.

Figure 1a,b illustrates the behavior of the samples of glass C and the glass–ceramic composite during heating from room temperature up to 1100–1330 °C. For glass C, the softening temperature is about 732 °C, the hemisphere formation is about 940 °C, the melting point (attributed to the point when the height of the sample shrinks to the one-third of the base) is 967 °C, and the temperature corresponding to intensive glass flow is 1100 °C. For the glass–ceramic composite, sample shrinkage begins at about 800 °C and ends at 1020 °C. Above this temperature, the sample tends to increase significantly in volume, indicating a reaction with the evolution of gaseous products.

This was confirmed in the studies of the microstructure of the samples, containing a significant fraction of spherical micropores, probably resulting from the entrapment of air in the sintered sample. The hemisphere formation of the glass–ceramic sample takes place at a temperature of about 1330 °C.

**Figure 2:**
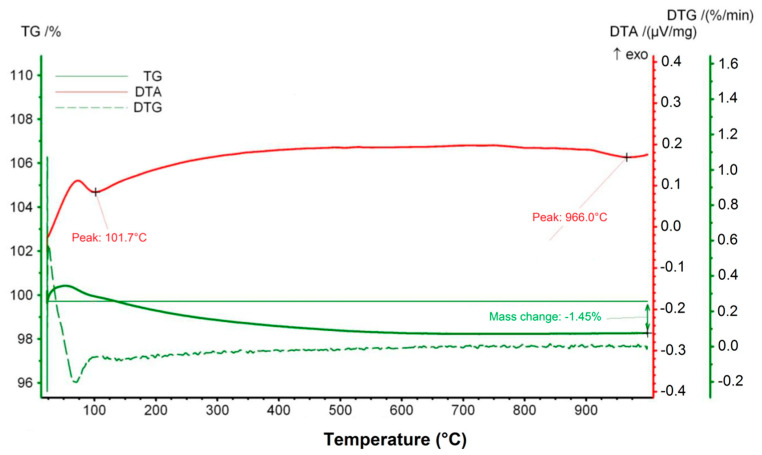
TG-DTG-DTA curves for the glass–ceramic composite.

Figure 2 presents the results of the thermal analysis (thermogravimetric TG—derivative thermogravimetric DTG—differential thermal analysis DTA) for the glass–ceramic composite under investigation. At a temperature of about 100 °C there occurs an endothermic peak on the DTA curve associated with the evaporation of absorbed moisture. Up to 600 °C, there is a slight monotonous weight loss (1.5%) and up to a temperature of about 400 °C, a slight evolution of heat. The further course of the DTA and TG curves up to 1000 °C is very stable. At 966 °C there is a weak endothermic effect associated with glass C melting. This temperature is consistent with the melting temperature determined based on heating microscope observations.

**Figure 3:**
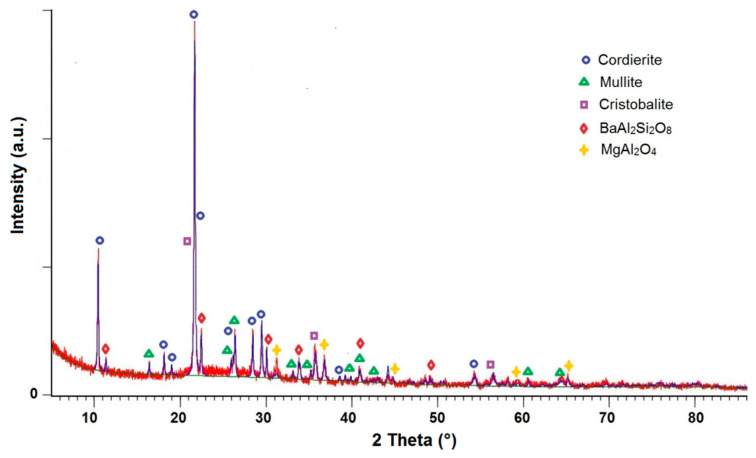
Diffraction pattern for the glass–ceramic composite sintered at 1020 °C.

Figure 3 shows a diffractogram of the glass–ceramic composite sintered at 1020 °C. Besides cordierite and mullite introduced as the crystalline phases, other crystalline phases were found in the sample, namely cristobalite, spinel MgAl_2_O_4_ and celsian BaAl_2_Si_2_O_8_. These phases are the result of crystallization from the glass phase—from SiO_2_ glass in the case of cristobalite and from glass C containing BaO, MgO and Al_2_O_3_ in the case of celsian and spinel.

**Figure 4:**
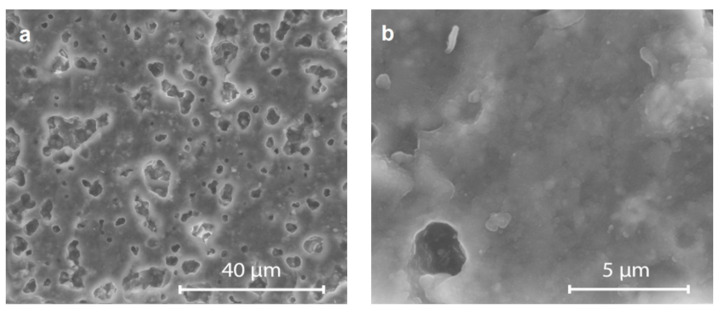

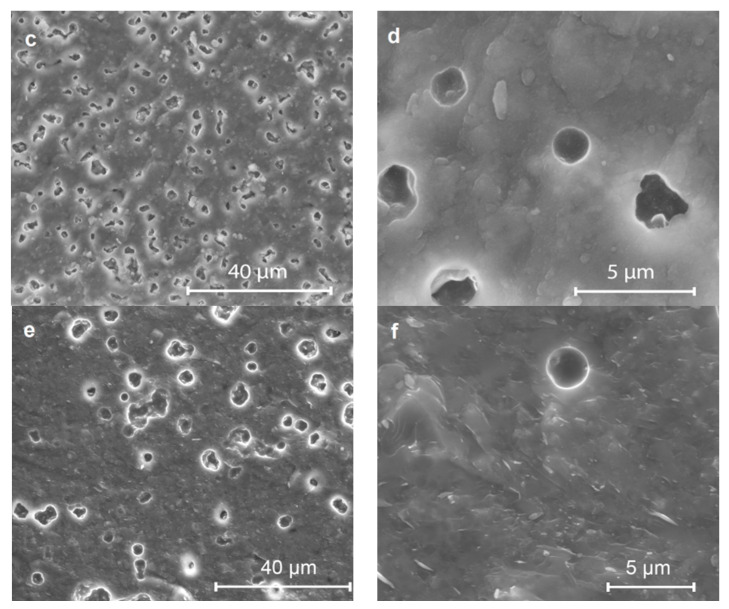
SEM images for the glass–ceramic composites, magnification, 3000 and 20,000. (**a**,**b**) sintered at 970 °C, (**c**,**d**), sintered at 1020 °C, (**e**,**f**) sintered at 1050 °C.

Figure 4 presents SEM images of the glass–ceramic substrates sintered at different temperatures. The images revealed a high glass phase content and a significant fraction of isolated spherical closed pores (10–25%), with diameters of 2–20 μm.

As illustrated in Figure 4b,d,f, in the areas between the pores the degree of densification is very high. Due to the large fraction of the glass phase, it is difficult to distinguish crystalline phase grains in the SEM images and using the EDS method. For the samples sintered at 1020 °C, the pores were smaller and more uniformly distributed than for those sintered at 970 °C and 1050 °C.

A few reasons may contribute to the pore formation observed: (1) the crystallization effect which impedes the progress of the sintering process, confirmed by the XRD analysis, (2) the significant fraction of SiO_2_ glass with high viscosity at the sintering temperatures used, (3) the insufficiently high fraction of glass C which shows a lower softening temperature and lower viscosity than SiO_2_ glass. The volume fraction of the low melting glass C in the glass–ceramic composite was 25% and that of the SiO_2_ glass—35%. The sintering conditions strongly influenced the microstructure, especially the porosity. It was stated that prolonging the sintering time (4–12 h) and enhancing the heating rate did not markedly improve the degree of densification. The effect of increasing the sintering temperature in the range 970–1050 °C was also not pronounced.

The most advantageous microstructure with about 10% of closed porosity was attained for the firing profile with a holding step at temperature 870 °C, followed by a final sintering at 970–1020 °C. The temperature of 870 °C chosen as a holding step corresponds to the temperature of the sphere formation of the low melting glass C, as revealed by the heating microscope observations. The lack of a low temperature step during the firing process of the glass–ceramic composites resulted in small open porosity at a level of 2–7%, even for the samples sintered at the highest temperatures.

### 3.2. Dielectric Properties

**Figure 5:**
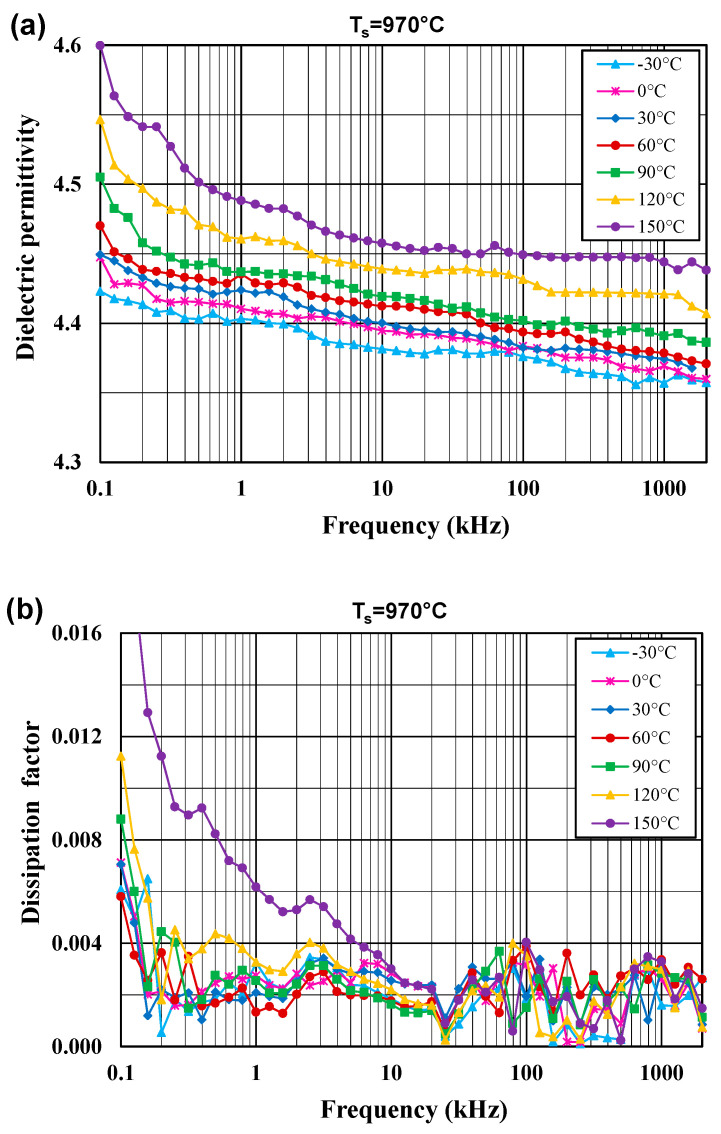
Dielectric permittivity (**a**) and dissipation factor (**b**) for the glass–ceramic composites sintered at T_s_ = 970 °C in the frequency range 100 Hz–2 MHz for a few temperatures in the range from −30 to 150 °C.

**Figure 6:**
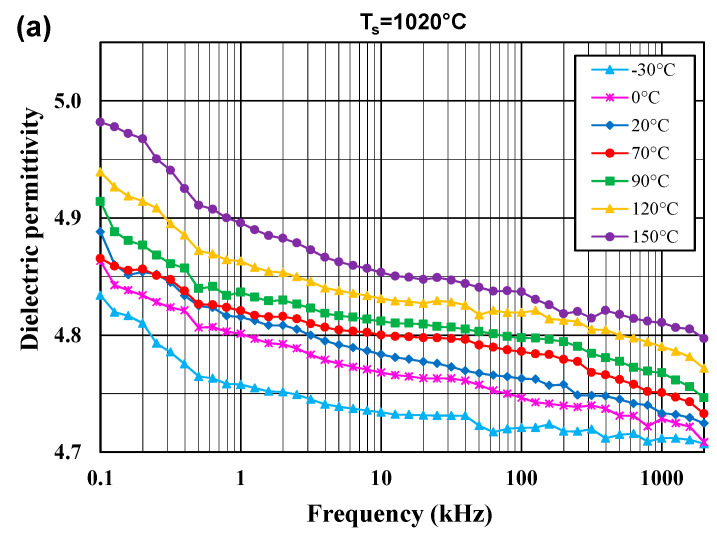

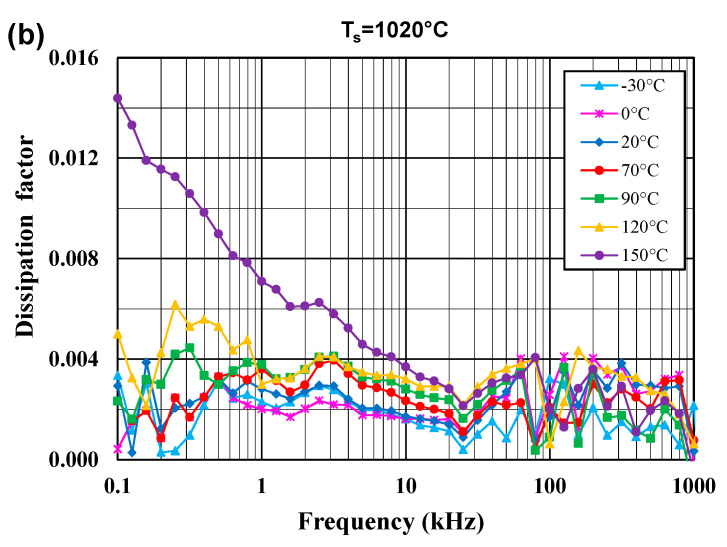
Dielectric permittivity (**a**) and dissipation factor (**b**) for the glass–ceramic composites sintered at T_s_ = 1020 °C in the frequency range 100 Hz–2 MHz for a few temperatures in the range from −30 to 150 °C.

Figure 5 and Figure 6 show the dielectric permittivity and the dissipation factor for the glass–ceramic composites, sintered at 970 °C and 1020 °C, in the frequency range 100 Hz–2 MHz and in the temperature range −30 to 150 °C.

The dielectric permittivity values slightly decrease with increasing frequency and with decreasing temperature. The enhanced values of the dielectric permittivity with rising measurement temperature can be attributed to thermally activated processes of the ionic conduction in glass and space charge, orientation and ionic polarizations in glass–ceramic composites, which are active in the low frequency region. For the Hz–MHz range, the dielectric permittivity of the samples sintered at 970 °C and 1020 °C assumes low values between 4.4 and 5.0. A higher sintering temperature leads to a slight enhancement of the dielectric permittivity due to the better degree of densification. The dissipation factor does not exceed 0.004 for lower temperatures and higher frequencies.

In Table 1 dielectric parameters determined in the frequency range 90–140 GHz are compared with the results obtained in the 100 Hz–1 MHz and 0.15–1.1 THz ranges. For the GHz range, only the samples sintered at the optimal temperature of 1050 °C were shown, while for the 100 Hz–1 MHz and 0.15–1.1 THz ranges besides the results for the optimal 1020–1050 °C sintering temperatures, the values for the lowest studied sintering temperature of 970 °C were also presented. For the samples sintered at 1050 °C, dielectric permittivity in the GHz band is 4.75–4.78, and the dissipation factor is 0.003–0.010. It follows from the comparison of the samples sintered at 1020–1050 °C that the values of dielectric permittivity are close in all three measured ranges with a slight tendency to decrease with frequency. However, an increase of dissipation factor with frequency rising from the Hz range to the THz range is significant.

**Figure 7:**
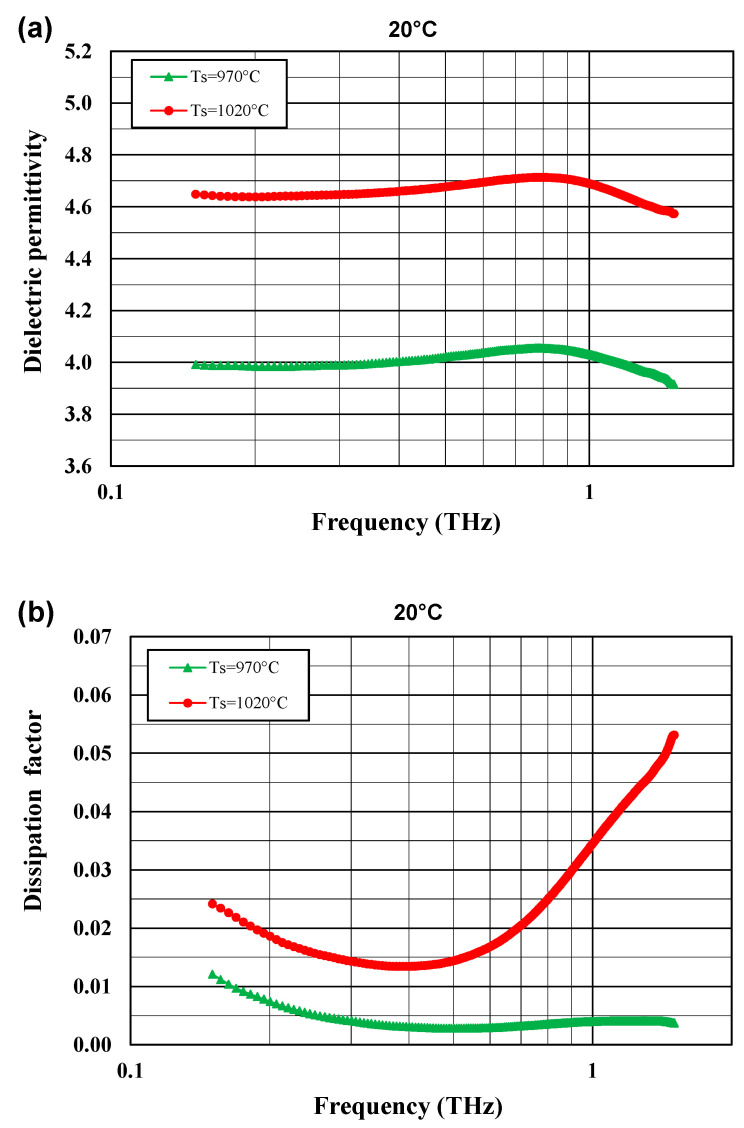
Dielectric permittivity (**a**) and dissipation factor (**b**) at 20 °C for the glass–ceramic composites sintered at T_S_ = 970 °C and T_S_ = 1050 °C as a function of frequency in the range 0.15–1.5 THz.

Figure 7a,b depicts the relationship between the dielectric permittivity and the dissipation factor at room temperature in the frequency range 0.15–1.5 THz for the glass–ceramic composite sintered at two temperatures, 970 and 1050 °C. The dielectric permittivity is very low in the 0.15–1.1 THz range and changes slightly with frequency. It assumes values of 4.0–4.1 for a sample sintered at 970 °C and 4.6–4.7 for a sample sintered at 1050 °C. For a sample sintered at 970 °C, the dissipation factor at 1 THz is 0.004 and is similar to that at 1 MHz, while for a sample sintered at 1050 °C the dissipation factor at 1 THz is 0.035 and is much higher than at 1 MHz.

The sintering temperature has a very significant impact on the dielectric permittivity and dissipation factor. Both the dielectric permittivity and the dielectric loss are distinctly lower for the samples sintered at a lower temperature. The number and distribution of pores as well as the presence of amorphous phases and secondary crystalline phases, strongly dependent on the sintering conditions of the glass-ceramic composites, are the factors influencing the dielectric properties observed.

The dielectric permittivity measured in the terahertz range is slightly lower than that in the Hz–MHz range and close to the value for the gigahertz range and almost frequency independent. These effects are consistent with the theoretical expectations of the model of damped harmonic oscillators, valid below the visible range [33,34]. At low frequencies, space charge polarization, orientation and ionic polarizations prevail, while at GHz and THz frequencies atomic and electronic polarizations are dominant and the real part of the dielectric permittivity attains a constant high-frequency value. Furthermore, porosity is a well-known crucial reason for the diminished dielectric permittivity because the dielectric permittivity of air trapped in the pores is close to 1.

It is worth noting that for the samples sintered in the same temperature of 1050 °C, the dissipation factor of the glass–ceramic composites in the THz range were higher than those in the GHz band. Such an effect was also reported by other authors [35] for a few substrate materials. For Al_2_O_3_ and Si_3_N_4_ polycrystalline samples, tan δ (dissipation factor) determined by the TDS method was two times higher at 1 THz than at 200 GHz. For two commercial LTCC materials, Ferro A6M, which is a highly crystallized glass–ceramic system, and DuPont 951, which is a glass–ceramic composite, this increase of tan δ with frequency is much higher (4–5 times) (0.003 and 0.019 at 200 GHz, and 0.012 and 0.097 at 1 THz for Ferro A6M and DuPont 951, respectively) [35].

Intrinsic dielectric losses are associated with the interaction of the phonon system determined by the crystal lattice structure with electromagnetic field. According to the damped harmonic oscillators theory [33,34] which describes complex dielectric permittivity as a function of frequency, the imaginary part of dielectric permittivity *ε″* and accordingly the dissipation factor are proportional to the frequency in the range much below the phonon frequencies (f < 1 THz). At very high frequencies, in the case of single phase and dense ceramics the intrinsic dielectric losses determined by the factors related to the crystal lattice structure, such as ionic polarizability, packing fraction, bond valence, should prevail and increase linearly with frequency according to the model of damped harmonic oscillators [33,34,36,37,38]. However, for microwave and submillimeter wave region, it was also stated [33] that along with intrinsic losses related to multiphonon absorption, extrinsic losses associated with defect induced one-phonon absorption can occur. For the glass–ceramic composites under investigation, the role of the extrinsic losses cannot be neglected, not only at low frequencies but also at high frequencies. The presence of the secondary crystalline phases and glassy phases, and the dumping of phonons at numerous interphases between the crystalline and amorphous phases, are supposed to be responsible for the relatively high dielectric losses of the materials under investigation.

## 4. Conclusions

Glass–ceramic composites prepared based on cordierite, mullite, silica glass and SiO_2_-B_2_O_3_-Al_2_O_3_-BaO-ZrO_2_ glass show very good microwave dielectric properties and can be sintered at relatively low temperatures of 970–1020 °C, feasible for cofiring with commercial Ag-Pd pastes in LTCC multilayer substrates. The glass–ceramic composites contain a significant fraction of spontaneously formed closed porosity (10–25%), strongly dependent on the firing profile. TDS studies in the frequency range 0.15–1.5 THz revealed that these substrates have a low dielectric permittivity at a level of 4.0–4.7, advantageous for submillimeter wave applications.

## Figures and Tables

**Table 1:** Comparison of dielectric properties in three frequency ranges for glass–ceramic composites sintered at T_s_ temperatures.

Dielectric Parameters at 20 °C	Frequency				
	100 Hz–1 MHz		90–140 GHz	0.15–1.1 THz	
Dielectric permittivity	4.36–4.43 (T_s_ = 970 °C)	4.72–4.89 T_s_ = 1020 °C)	4.75–4.78 (T_s_ = 1050 °C)	4.0–4.05 (T_s_ = 970 °C)	4.65–4.71 (T_s_ = 1050 °C)
Dissipation Factor	0.002–0.007 (T_s_ = 970 °C)	0.002–0.004 (T_s_ = 970 °C)	0.003–0.010 (T_s_ = 1050 °C)	0.003–0.012 (T_s_ = 970 °C)	0.013–0.039 (T_s_ = 1050 °C)

## Data Availability

The data presented in this study are available on request from the corresponding author. The data are not publicly available as the data also form part of an ongoing study.
